# Supplementary material for: The Risk of Invasive Pneumococcal Disease Differs between Risk Groups in Norway Following Widespread Use of the 13-Valent Pneumococcal Vaccine in Children
Source: Microorganisms. 2021 Aug 20;9(8):1774. doi: 10.3390/microorganisms9081774 (PMC8398338; doi:10.3390/microorganisms9081774)
Supplement: Supplementary file 1 [file microorganisms-09-01774-s001.zip › microorganisms-1303867-supplementary.pdf]

Supplementary material

Supplementary Table S1 : Overview of the included data, the data sources and the percentage of cases with available data

Variable	Kind of data	Data source	Years included	% of cases with data
Index date / year	Notification data, date of sampling of material for pneumococcal identification	MSIS	2009-2017	100%
Demographic information of the IPD case	Notification data	MSIS	2009-2017	100%
Serotype	Notification data / laboratory data	Reference laboratory NIPH	2009-2017	97.6%
Underlying medical risk conditions	Hospital discharge data, ICD-10 codes	NPR	2008-2017	100%
Vaccination history	Registry data	SYSVAK	2009-2017	High coverage for children born in 2006 or later, unknown coverage for those born before 2006
	Physicians	Letter/phone calls	2015-2017	Total 98%

Supplementary Table S2: ICD-10 codes used to categorise medical risk conditions

Medical risk condition	ICD-10 code
High-risk conditions (i.e. immunosuppressive conditions)	
Congenital/acquired immunodeficiency including human immunodeficiency virus (HIV) disease and functional or anatomical asplenia	D80-D89, B20-B24, D56-D57, D58.2, D73
Chronic kidney disease including nephrotic syndrome	N04, N18
Haematological cancer (including leukaemia and lymphoma)	C81-C85, C90-C93, C96.9
Generalized malignancy (Metastatic solid tumours)	C76-C80
Organ and tissue transplantation	Z94
Medium risk conditions	
Cerebrospinal fluid (CSF) leakage or cochlear implant	G96.0, Z96.2
Chronic heart disease	I25, I42, I50
Chronic lung disease	J43-J45
Diabetes mellitus	E10-E14
Chronic liver disease including cirrhosis	K72-K74, K76, C22
Other malignancies	C00-C75
Alcoholism	F10, G31.2, G62.1, G72.1, I42.6, K29.2, K70, K85.2, K86.0, O35.4

Supplementary Material S3: Sensitivity analysis on the use of immunosuppressive treatment among those with and without medical risk conditions.

Iatrogenic immunosuppression is a risk factor for invasive pneumococcal disease. In the work presented in the main manuscript, we did not have information on the use of immunosuppressive treatment. In this sensitivity analysis we aimed I) to determine the proportion of IPD cases with no defined medical risk conditions that uses immunosuppressive treatment, as indication of the size of misclassification of those with increased risk to IPD that are defined as no additional risk for IPD, and II) to describe the use of immunosuppressive treatment in the defined medical risk groups.

Methods

Data used in this sensitivity analysis comes from another study that has been published by Steens etal (1) and includes all notified IPD cases in Norway in the period 2009-2014. Because of the available ethical clearances it was not possible to link the data to the data used in the main manuscript, though, the original data (the included case population) are the same for that period.

For all IPD cases, ICD10-codes obtained from NPR were used to define medical risk conditions as described in the main manuscript, except for the following codes/conditions that could not be included:

- ICD10 code D58.2 could not be included in the category Functional or anatomical asplenia
- ICD10 code C96.9 could not be included in the category Leukaemia
- ICD10 codes K85.2 and O35.4 could not be included in the category Alcoholism

Data on prescriptions dispensed in ambulatory care defined by Anatomical Therapeutic Chemical (ATC) codes were obtained from the Norwegian Prescription Database. We defined the use of immunosuppressive treatment as having a dispensed prescription during the last 12 months of chemotherapy (ATC code L01), long term use of systemic corticosteroids (ATC codes H02A and H02B) or other immunosuppressants (ATC code L04A). More details about the classification and analyses of the prescription data can be found in reference (1).

Results

Of the 4046 IPD cases that were notified in 2009-2014, 87.4% had a medical risk condition defined based on ICD10 codes and 17.3% used immunosuppressive treatment.

Of those defined as not having a medical risk condition, 4.9% used immunosuppressive drugs that increase the risk for IPD (1). For age-group specific numbers, see Supplementary Table S3. Those without defined medical risk condition based on ICD10 codes but that use immunosuppressive treatment mainly used long term treatment with systemic corticosteroids (88%) or other immunosuppressants (36%). Few cases without medical risk conditions used chemotherapy in ambulatory care (4%).

Table S4_1: Percentage of IPD cases in 2009-2014 with medical risk conditions, on immunosuppressive treatment or both/none

Age group	Number of cases (%)	% with risk conditions defined on ICD-10 codes	% using immunosuppressive treatment based on ATC codes	% without risk conditions based on ICD10 codes but on immunosuppressive treatment
<20 years	258 (6.4)	67.8	5.8	0.0
20-49 years	664 (16.4)	86.1	11.3	4.3
50-64 years	1031 (25.5)	87.0	17.0	7.5
65-74 years	826 (20.4)	91.4	26.0	4.2
75-84 years	762 (18.8)	91.1	21.1	7.4
≥85 years	505 (12.5)	87.7	11.5	4.8
Total	4046 (100)	87.4	17.3	4.9

Of the cases with a medical risk condition based on the defined ICD-10 codes, 19.1% used immunosuppressive treatment. Of them, 79.2% used long term treatment with systemic corticosteroids, 32.6% other immunosuppressants and 24.0% used chemotherapy. The percentage that uses immunosuppressive drugs among the different medical risk groups are presented in Supplementary Table S5.

Table S4_2: Percentage of IPD cases in 2009-2014 that use immunosuppressive treatment per medical risk condition. Note that people may use more than one kind of immunosuppressive drug, and that the sum of corticosteroids, chemotherapy and other immunosuppressants may be larger than the percentage using any immunosuppressive drug.

Underlying risk condition	Number of cases in risk group	% using any immunosuppressive treatment	% using corticosteroids	% chemotherapy	% other immunosuppressants
Congenital/acquired immunodeficiency	65	41.5	29.2	9.2	20
Chronic kidney disease	280	35.7	31.1	6.4	18.6
Haematological cancer	362	53.0	34.0	23.2	27.9
Generalized malignancy	219	48.4	39.3	23.7	3.2
Other malignancies	546	32.2	25.8	13.9	2.8
Organ and tissue transplantation	72	87.5	59.7	2.8	84.7
CSF leakage or cochlear implant	12	8.3	8.3	0.0	0.0
Chronic heart disease	645	23.3	20.9	4.3	4.7
Chronic lung disease	769	32.1	29.3	4.6	4.9
Diabetes mellitus	451	23.7	20.4	4.0	7.3
Alcoholism	131	8.4	7.6	1.5	3.1
Chronic liver disease	95	25.3	16.8	4.2	13.7

When looking at the data the other way around, among those on immunosuppressive treatment, the most common medical risk conditions were chronic respiratory disease (35.3%), haematological cancer (27.5%), chronic heart disease (21.5%), diabetes mellitus (15.3%) or chronic renal disease

(14.3%). The other conditions covered less than 10% of the IPD cases on immunosuppressive treatment.

Conclusions

By defining medical risk groups only on ICD-10 codes we miss 4.9% as medical risk group (namely, on immunosuppressive treatment; called misclassified). Misclassification was most common among those aged 50-64 or 75-84 years (almost 7.5%). Those we misclassified as not being in a medical risk group for IPD mainly used long term treatment with systemic corticosteroids or other immunosuppressants. The majority that uses chemotherapy in ambulatory care (the immunosuppressive treatment that is associated with the highest risks for IPD (1)) have a medical risk condition for which they have a specialist counselling at least every two years, which will have been defined by ICD10 codes and were therefore categorised as having a medical risk condition. Overall, the misclassification will therefore only have a minor effect on the results presented in the main manuscript.

Of those with medical risk conditions, one fifth also uses immunosuppressive drugs that have been shown to increase the risk to IPD. The percentage on immunosuppressive treatment was highest among those with a history of organ or tissue transplantation, followed by those with haematological cancer, generalised malignancy and congenital/acquired immunodeficiency.

References

1. Steens A, Winje BA, White RA, Odsbu I, Brantsaeter AB, Vestrheim DF. Indirect effects of pneumococcal childhood vaccination in individuals treated with immunosuppressants in ambulatory care: a case-cohort study. Clinical Infectious Diseases. 2018.
